# IFNγ in human sepsis: a scoping review

**DOI:** 10.1186/s13613-025-01534-z

**Published:** 2025-08-05

**Authors:** Daniel Thomas-Rüddel, Evangelos Giamarellos-Bourboulis, Caroline Neumann, Josef Briegel, Antoine Roquilly, Djillali Annane, Reinhard Wetzker, Michael Bauer, for the iRECORDS study group, Sara Aly Abdelghany, Maha Khalaf AlyAly, Mohamed Gamal Elansary, Shereen Mustafa Elgengeehy, Heba Mostafa Elwi, Yasser Sadek Nassar, Rania Yehia Hash, Rania Bouneb, Zaineb Chelly-Dagdia, Katy Diallo, Jérome Fleuriet, Henri-Jean Garchon, Stanislas Grassin Delyle, Rahma Hellali, Nicholas Heming, Nicolas Hunzinger, Elodie Lamy, Jihene Mahmoud, Virginie Maxime, Pierre Moine, Camille Roquencourt, Marie Alice Vovy, Karine Zeitouni, Manuela Adling-Ehrhardt, Frank Bloos, Sandra Frank, Katharina Habler, Ludwig Hinske, Rainer König, Dorothea Lange, Margit Leitner, Marcus Oswald, Christina Scharf-Janssen, ichael Vogeser, Carlos Flores, Jesús Villar

**Affiliations:** 1 Department of Anesthesiology and Intensive Care Medicine, Jena University Hospital, Am Klinikum 1, 07747 Jena, Germany; 2 4th Department of Internal Medicine, Medical School, National and Kapodistrian University of Athens, Athens, Greece; 3 Department of Anesthesiology, University Hospital, LMU Munich, Munich, Germany; 4 Center for Research in Transplantation and Translational Immunology, UMR 1064; and Anesthesie Reanimation, Nantes Université, CHU Nantes, INSERM, CIC 1413, Nantes, France; 5 Department of Intensive Care, Raymond Poincaré Hospital, APHP, University Versailles Saint Quentin–University Paris Saclay, INSERM, Garches, France

**Keywords:** Sepsis, IFNγ, Hyperinflammation, Immunosuppression, Immunosuppressive therapy, Immunostimulatory therapy

## Abstract

**Background:**

The cytokine IFNγ is released primarily by lymphocytes to initiate and orchestrate immune responses in a broad range of target cells. Whereas immune cells release inflammatory mediators and initiate antimicrobial responses when stimulated by IFNγ, parenchymal cells frequently display increased immunogenicity and incidental cell death. In addition to these well-characterized effects of IFNγ, recent studies disclose a key role of the cytokine in sepsis and organ dysfunction.

**Main:**

This review summarizes current knowledge on the IFNγ response to infection and attempts to relate the IFNγ response to endophenotypes of sepsis in the human host. Both, excessive pro-inflammatory responses with high IFNγ and downstream mediators, such as chemokines (CXCL9), as well as immunosuppression with low IFNγ levels are associated with unfavorable outcomes in sepsis. Pilot studies suggested beneficial effects of recombinant IFNγ in counteracting immunosuppression associated with low IFNγ levels. On the other hand, IFNγ may induce macrophages to release chemokines CXCL9, 10, and 11 to attract B and T lymphocytes to the sites of infection. Downstream induction of CXCL9 (but not of CXCL10 and 11) occurring in a subset of patients with high IFNγ levels has been shown to correlate with the hyper-inflammatory phenotype of sepsis. Both, high- and low-expressing IFNγ phenotypes of sepsis, might be related to nucleotide polymorphisms of the human IFNγ gene.

**Conclusion:**

Association of IFNγ activity states with sepsis outcome renders this key regulatory protein of immunity a top candidate for theranostic interventions in a “personalized medicine approach” to infection and sepsis, especially when combined with additional biomarkers, such as CXCL9, reflecting or even mediating maladaptive downstream actions.

**Graphical Abstract:**

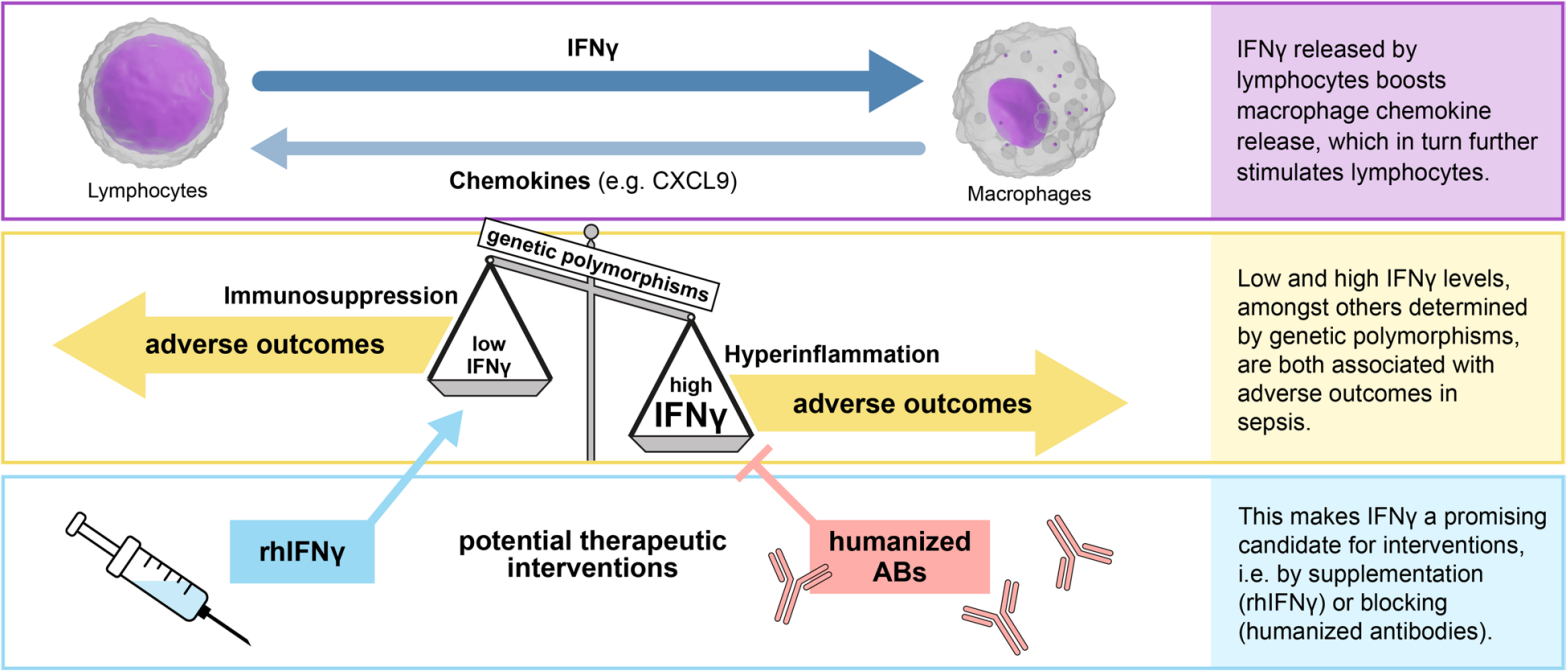

## Background

Together with the emergence of first vertebrates 500 million years ago, the crude innate immune responses of lower eukaryotes to pathogens have been complemented by “adaptive” immune reactions exhibiting high specificity and memory traits [1]. Proper functioning of the advanced vertebrate immune system required efficient coordination of the humoral and cellular elements of innate and adaptive immunity. The cytokine interferon gamma (IFNγ) plays a key role in the underlying regulatory network [2].

**Fig. 1:**
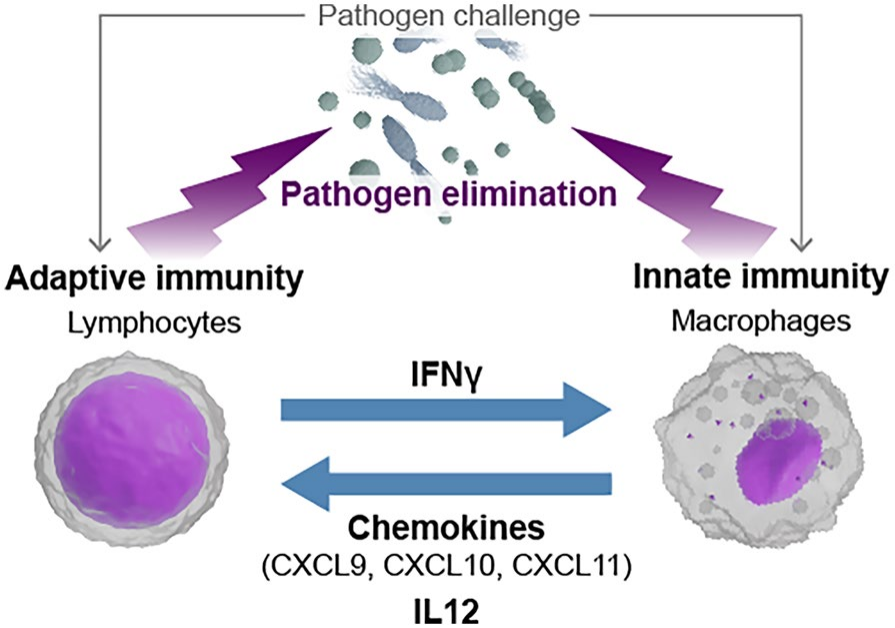
IFNγ and downstream cytokines and chemokines implicated in innate and adaptive immune responses to infection. IFNγ and several downstream cytokines and chemokines act as main players of the cooperative immune response to infection. After initial contact of tissue macrophages and resident T cells, lymphocytes release IFNγ, which boosts innate resistance responses of macrophages. These responses include secretion of the cytokine IL-12 and of chemokines, which are aimed to attract and to further stimulate lymphocytes. In a self-accelerating circuit the overall immune response further increases (ideally) until complete elimination of invading pathogens

Experimental studies in vitro and in vivo identified lymphocytes, the main cellular entity of adaptive immunity, as the main source of IFNγ [2]. IFNγ was found to be released by T-cells and B-cells after stimulation by pathogens which are presented by professional antigen-presenting cells and by pro-inflammatory cytokines released by myeloid cells [3]. In addition, innate lymphoid NK cells after “missing self-recognition” have been found to release IFNγ [4]. IFNγ released by activated lymphocytes has been originally identified as a key mediator of monocyte and macrophage functions [5]. Basically, all major functions of these innate immune cells can be affected by the cytokine. This includes production of bactericidal oxygen radicals, migration, phagocytosis of pathogenic microorganisms and release of additional humoral immune mediators, such as cytokines and chemokines (Fig. 1) [6, 7]. Parenchymal cell functions affected by IFNγ include proliferation, apoptosis and secretion [5, 6]. Innate immune cells, such as macrophages, as well as parenchymal cells express specific IFNγ receptors. Corresponding downstream signaling proteins include the Janus tyrosine kinases JAK1 and JAK2 and so-called signal transducers and activators of transcription (STATs) [8].

One of the key responses initially induced by IFNγ in macrophages is the release of the chemokines CXCL9, CXCL10 and CXCL11, which attract additional T-lymphocytes and other immune cells from the bloodstream to the focus of infection (Fig. 1) [7]. These chemokines and a series of cytokines, represented here by the prototypical IL-12, are secreted by macrophages, cause further stimulation of IFNγ producing lymphocytes leading to a cooperative self-accelerating immune response circuit between lymphocytes and macrophages as the key representatives of adaptive and innate immunity [9, 10].

Considering the predominant role of IFNγ as a stimulator of innate immune cells, significant pathological effects of dysregulated IFNγ levels seem plausible. Indeed, IFNγ deficiency has been shown to induce a pronounced increase of susceptibility to infection. A prominent example for hereditary disease with impaired IFNγ activity is Mendelian susceptibility to mycobacterial disease (MSMD) but other entities characterized by low production or decreased activity of IFNγ or its receptors and downstream signaling mediators like STATs are also described [2, 9]. Significant progress in the treatment of these immunodeficiency-related diseases has been achieved with the application of recombinant IFNγ protein [2, 11], supporting its role as a candidate immunostimulatory drug for a personalized approach in the critically ill [12].

In contrast to increased susceptibility to infection induced by suboptimal activity of IFNγ, patients with IFNγ overproduction exhibit typical hyperinflammatory disorders, such as lupus erythematodes and hemophagocytic lymphohistiocytosis (HLH), including macrophage activation syndrome [13], the latter reflecting a rare but well defined endotype of sepsis. Interferon therapy can, similar to its role in autoimmune disease, cause a range of off-target effects related to its (intended) immune-stimulatory action, such as flu-like symptoms and fatigue, cognitive and mood changes, and altered organ function e.g. in the gastrointestinal tract. Of note, these effects can augment an ongoing inflammatory process in the septic host [14] as will be discussed in detail below.

Notably, current understanding of sepsis can involve either IFNγ suppression or hyperactivity [11, 15–17]. In this scoping review, we explore the correlation of IFNγ levels to sepsis endotypes and put these into perspective of the knowledge on chronic states of altered IFNγ signaling. The review also presents recent insights validating IFNγ-induced immune responses as a target for therapeutic intervention.

## Method

**Fig. 2:**
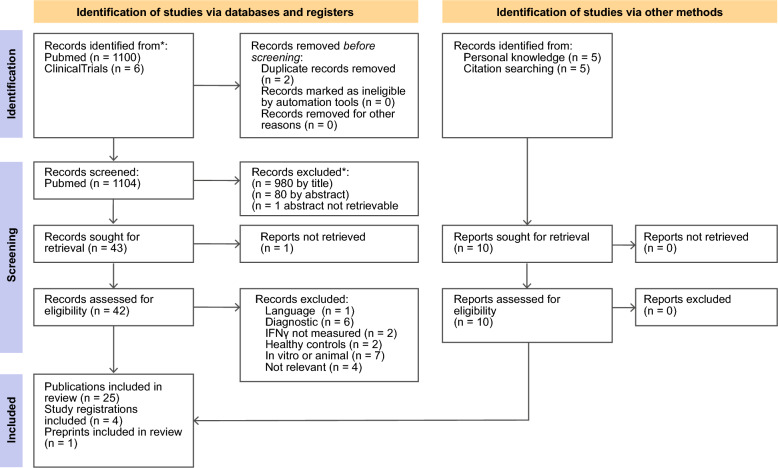
PRISMA flow diagram of selection of sources of evidence

The significance of IFNγ in sepsis has been explored systematically by a PUBMED search for “("Sepsis"[Mesh]) AND "Interferon-gamma"[Mesh]” on 8th of August 2024. All search results were screened in three steps for eligibility, titles only first, then abstracts and in a final step full texts after retrieval. All steps were performed by a single researcher (DTR). We included papers that reported on human patients and either the effects of IFNγ therapy in sepsis, a prognostic role of IFNγ in sepsis, an identification of sepsis subtypes by IFNγ levels or the role of IFNγ in the risk for developing sepsis or the use of IFNγ for sepsis prevention. We did not include studies that assessed IFNγ production in vitro. The initial screening resulted in 1100 potentially relevant records found in PubMed and were screened for inclusion in this review as detailed Fig. 2.

## Results and discussion

Current concepts suggest that sepsis immune phenotypes can be characterized by predominant hyper-inflammation or immunosuppression [18, 19]. We searched for connections of these phenotypes with the key immune effector molecule IFNγ in sepsis patients. The related references include assessment of IFNγ levels in phenotypically characterized sepsis patients, investigations of the correlation of polymorphisms in the IFNγ gene with sepsis phenotypes and attempts to treat failing immune responses and immunosuppression by therapeutic application of recombinant IFNγ [summarized in Table 1].

**Table 1:** Summary of selected clinical studies describing observational as well as interventional studies, which involve IFNγ as a diagnostic or therapeutic target and with respect to underlying clinical phenotype

Short description and citation	Patient population	Patient numbers	Study design	Summary of outcomes	Suscepti-bilility to infection	Immune pathology
IFNγ as a diagnostic marker						
Restauration of monocyte function in septic patients [11]	Patients with sepsis (II)	9	Pilot trial: treatment of septic patients with HLA-DR^+^ <30% of monocytic cells with 100 µg IFNγ per day (s.c.) Endpoint: positivity of >50% monocytes HLA-DR^+^ for 3 consecutive days	Improvement of lipopolysaccharide TNFα response in 8 of 9 patients; Recovery in 7 patients from sepsis with significant improvement of organ dysfunction score	X	
Risk stratification of immunosuppressed endotypes with adverse outcome [16]	Patients with sepsis (III)	107	Prospective, observational multicentre trial in comparison to 68 non-septic ICU controls and 46 healthy controls Longitudinal design (days 1, 4 and 7) Endpoints: long term mortality rate (180 days)	Discrimination of later non-survivors by determination of cellular IFNγ release analysed *ex-vivo* by whole-blood immunosorbent assay	X	
Endotype classification of IFNγ-driven sepsis (IDS) [28]	Patients with sepsis (III)	5503	Discovery set: 3670 patients Validation set: 1833 patients Endpoints: frequency of IDS as independent sepsis endotype associated with 28-day mortality (primary) Association of over-time changes of IDS endotype with 28-day outcome (secondary)	Differentiation IDS from macrophage activation-like syndrome Identification of IDS as an independent risk factor for death in presence of other endotypes, severity and organ dysfunction	X	
Decreased PBMC-expression of IFNγ and TNFα is associated with unfavourable outcome [21]	Patients with sepsis/septic shock (II)	62	Monocentric observational trial (day 1, day 7) Disease controls: Bloodstream infection with gram^−^ bacteria w/o organ failure, healthy controls Endpoints: development/persistence of septic shock; mortality rate	Expression profile of IFNγ (decrease) is associated with sepsis/sepsis shock; Low expression of IFNγ and TNFα is associated with shock, severity and unfavourable outcome at day 7; no correlation with circulating protein levels	X	
Increased levels of IFNγ indicates risk for secondary Candida infection [25]	Patients with sepsis (III)	31	Monocentric observational study 19 patients with sepsis 12 patients with sepsis/secondary Candida infection Endpoints: IFNγ	Exogeneous IFNγ suppressed macrophage phagocytosis of zymosan, blockade improved survival of secondary candidemia following endotoxinemia	X	
INFγ ratio to IL10 for risk stratification of septic shock patients for hydrocortisone treatment [26]	Patients with septic shock (III)	467	Multicentric interventional trial (CORTICUS) Hellenic sepsis study group (HSSG) 83 patients for explorative analysis (CORTICUS) 329 patients for validation (HSSG etc.) Endpoints: survival rate	IFNγ/IL10 ratio identified as a molecular marker supporting the decisions for administration of hydrocortisone in patients with septic shock	X	
Molecular fingerprinting of resistance towards pathogens and systemic inflammation [29]	Patients with septic shock (III)	125	Monocentric observational study (FUSE) Comparison of datasets obtained in eight other trials Endpoints: 28d-survival rate	Clinical phenotype is reflected in protein and gene expression profile allowing sub-classification with respect to impaired balance between resistance and systemic inflammation prediction short-term survival rate	X	
Functional comparison of four IFNγ-polymorphisms and its association with pneumonia-induced sepsis [60]	Patients with pneumonia induced sepsis	196	Observational trial 213 age- and sex-matched control subjects Endpoints: severity	Association with progression and outcome: +874 T as well as -1616TT genotype were found protective against development of severe sepsis		X
Genetic variants in a master regulator of IFNγ expression (STAT4) in non-thyphoidal Salmonella (NTS) bacteremia [61]	Patients with NTS bacteremia	180	Genome wide association study (2677 controls)	Context-specific expression quantitative trait locus for STAT4 expression is described as an NTS risk genotype resulting in decreased IFNγ expression either in natural killer cells as well as in circulation during bacteremia		X
IFNγ as a therapeutic target						
Reversal of immunoparalysis following endotoxinemia [36]	Administration of either *rh*IFNγ or *rh*GM-CSF	18	Double-blind placebo-controlled randomized study Endpoints: normalization of biochemical parameters	IFNγ-treatment attenuated ex vivo impaired TNFα release as a marker of immunoparalysis as well as normalized HLA-DR expression	X	
Adjuvant immune-stimulation in immunoparalysed septic patient with IFNγ [37]	Patient (58 yrs) with prolonged disseminated *Staph. aureus* sepsis	1	Case-report Endpoint: clinical recovery	Immuno-monitoring and gene expression profiling (antigen presentation, T-Helper cell function etc.) resolution of immune dysfunction	X	
Adjunctive immunotherapy in immunosuppressed patients [39]	Septic patients (II) with immunosuppression	20	Multicentric interventional trial Endpoints: normalization of HLA-DR expression	Adjunctive immunotherapy with IFNγ improved immune host defence in sepsis induced immune suppression	X	
Immuno activation targeted immunotherapy in patients with sepsis [40]	Septic Patients (III) with either macrophage activation-like syndrome (MALS), immune paralysis or mixed phenotype	240	Double blind, double-dummy randomized trial Endpoints: 28-day survival rate	Identification of three independent immune classification strata (MALS, paralysis or mixed), which are proposed as stratification for personalized adjuvant immunotherapy	X	
Administration of IFNγ for prevention of severe infection [41]	Patients with chronic granulomatous disease	128	Double-blind placebo contr.trial (IFNγ 50 µg/m^2^, 3x/s for 1y) Endpoints: Time to infection (i.e. hospitalization due to infection/administration of parenteral antibiotics)	Increase in time to infection and decrease of absolute numbers of infections in IFNγ treated group	X	
Prevention of hospital acquired pneumonia in ventilated patients by IFNγ [14]	Patients with organ failure (≥1) undergoing invasive ventilation	109	Multicentre, placebo-controlled randomized trial Endpoint: hospital acquired pneumonia (HAP)/all cause 28d-mortality (composite)	Early treatment with IFNγ resulted in an adjusted hazard ratio for HAP or death of 1.76 Discontinuation due to safety concerns	X	

Enrolled patients and inclusion according to sepsis-II or -III definitions and readout for outcome are indicated

Underline is used to mark the "endpoints" studied but can be ommitted

Our literature search suggests a clear assignment of high IFNγ levels to sepsis patients exhibiting the hyper-inflammatory phenotype, whereas a low or diminished IFNγ response is associated with the immunosuppressive endophenotype. Basic characteristics of both patient phenotypes will be now introduced in more detail.

### *IFN*γ* deficiency in immunosuppression*

The significance of the IFNγ response for the development of septic immunosuppression was originally shown in studies investigating IFNγ release of whole blood or isolated leukocytes from sepsis patients to agents stimulating T-cells. These studies revealed significant decrease of IFNγ production in nonsurvivors in comparison to healthy donors or surviving patients [16–20].

An exemplary comparative analysis of Barrios et al. [16] showed in addition to downregulation of IFNγ a decreased expression the MHC class II cell surface receptor HLA-DR on monocytes, i.e. a validated readout for immunosuppression in the critically ill, in nonsurviving patients indicating extensive loss of innate immune responses in the nonsurviving patients. Septic patients with low IFNγ expression were older and had lower absolute leukocyte counts and higher level of the immunosuppressive mediators PD-L1 and IL-10. Feasibly, these findings pave the way for novel treatment options of immunosuppressed sepsis patients using the clinically established “checkpoint” PD-L1 inhibitors or IL-10 antibodies for restoring T-cell functions presumably inducing restauration of the IFNγ response.

### *Increased IFN*γ* in septic hyperinflammation*

Increased IFNγ levels in blood were reported for bacteremia treated outside the ICU and for severe sepsis [21, 22]. High IFNγ levels were also observed in survivors of septic shock [23] or sepsis in general [21], while others reported no significant differences [24]. One study reported an association of high initial IFNγ levels with secondary Candida infections in the survival phase of bacterial sepsis [25]. The IFNγ/IL-10 ratio was suggested as a potential marker for a favorable response to hydrocortisone treatment [26, 27].

A recent clinical study reports a pro-inflammatory endotype of sepsis associated with high blood levels of IFNγ and increased mortality [28]. The endotype of IFNγ-driven sepsis (IDS) was defined as blood IFNγ levels above 3 pg/mL (associated with a low risk for immunoparalysis) and simultaneously increased CXCL9 levels (2200 pg/mL), a chemokine selectively induced by IFNγ in macrophages. While IDS has been identified as an independent risk factor for death in sepsis patients, decreases of IFNγ and CXCL9 blood levels within the first 72 h were associated with better outcome.

Together these studies reveal a frequent increase of IFNγ blood level in patients exhibiting the hyperinflammatory phenotype of sepsis. Another recent report claims a crucial role of the balance between transcriptional programs for regular “resistance” responses and pathological “systemic inflammation” in sepsis etiology [29]. IFNγ and the downstream chemokines CXCL10 and CXCL11 were identified as key biomarkers for the “resistance” pattern. Sepsis was characterized by a low resistance vs. high systemic inflammatory response, marked by low expression of IFNγ and the related chemokines [29]. Interestingly, the chemokines CXCL10 and CXCL11 seem to play the predominant role as mediators of appropriate antimicrobial resistance responses, in contrast to CXCL9.

The given studies suggest a predominant role of the chemokines CXCL9, CXCL10 and CXCL11 as “executors” of IFNγ functions, while the mechanistic background underlying their differential functionality remains unclear. All three chemokines are released by macrophages, dendritic cells or parenchymatous tissue following stimulation with IFNγ [10]. While CXCL9 expression requires IFNγ, CXCL10 and CXCL11 production are also stimulated by other type I and type II interferons [10, 28] indicating its candidate role to specifically mediate downstream actions of IFNγ. Induced by IFNγ in macrophages all three chemokines attract B and T lymphocytes to the sites of inflammation but only CXCL9 expression has been shown to correlate with severity of hyper-inflammatory states including the IDS endotype of sepsis [28]. Jointly these findings disclose CXCL9 as specific marker of sepsis pathology and presumably other hyper-inflammatory syndromes [10, 28].

Collectively these studies characterize IFNγ as a key mediator of infection-induced inflammatory responses in infectious diseases including sepsis.

The inducible expression of IFNγ displays high inter-individual variance, which is frequently caused by genetic polymorphisms. In the human IFNγ gene a single nucleotide T to A polymorphism in position 874 of the first intron appears of particular significance for expression control. This polymorphism is followed by a variable CA microsatellite sequence, which has been shown to affect IFNγ expression level via a putative NF-κB binding site of the gene [30]. The regulatory effects of the polymorphic CA repeat on IFNγ expression are tightly connected to the 874 T/A polymorphism. In fact, 874 TT, AA and AT genotypes are associated with high, low and intermediate production of IFNγ in vitro and in vivo [30, 31]. Majority of related studies indicate an increased susceptibility of the high IFNγ expressing TT genotype connected to allele 2 with 12 CA repeats to hyper-inflammatory syndromes and autoimmune diseases [32]. In contrast the low IFNγ expressing AA genotype occurs more frequently in patients suffering from immunosuppression and increased susceptibility to infections, particularly tuberculosis [31].

Despite these initial reports, the diagnostic potential of these polymorphisms in sepsis has not yet been exploited. These regulatory elements are especially promising for correlative analysis of sepsis endotypes and predictive enrichment for immune modulation among sepsis patients.

### *IFN*γ* as a target for treatment of sepsis*

**Fig. 3:**
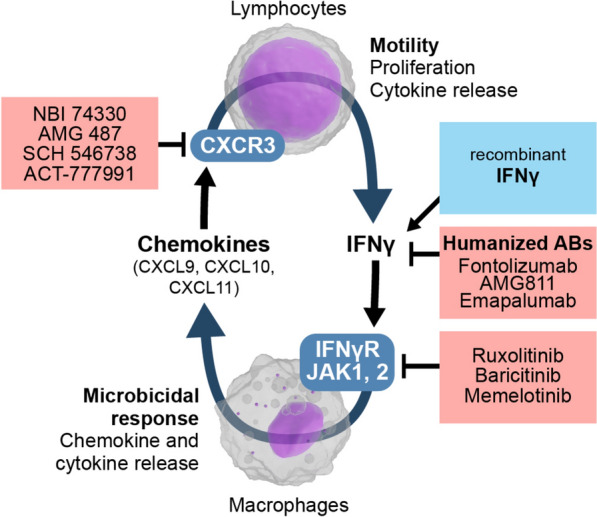
Prospects of modulation of IFNγ and its downstream signaling cascades in adjunctive therapy of sepsis. Topology of the IFNγ—chemokine signaling circuit between lymphocytes and macrophages and related treatment options for immunosuppression (blue) and hyperinflammatory (red) phenotypes of sepsis. Studies reporting use of recombinant IFNy are two case reports [37, 38] and in uncontrolled case series [11, 39, 40]. Results of a multicenter trial (ImmunoSep; NCT04990232) are currently unpublished. All other drugs have been only testet in animal experiments or preclinical settings

Analysis of IFNγ status in patients suffering from autoimmune diseases or infections unveils association of inflammatory responses with high IFNγ levels whereas low IFNγ goes parallel with a decline of antimicrobial defense. Consequently, treatment approaches have been developed for diseases induced by deviant levels of IFNγ (Fig. 3). Exemplarily, patients with inborn low level of IFNγ activity like MSMD have been successfully treated by compensatory therapy with recombinant IFNγ [33]. Initial findings also indicate improved immune host defense in patients exhibiting sepsis-induced immunosuppression by adjunctive immunotherapy with IFNγ [11, 34]. As IFNγ reflects a double-edged sword and increasing evidence supports a “compartmentalization” of (mal)adaptive immune responses, particularly in the lungs, alternative routes to administer IFNγ, such as inhalation, might be promising to prevent off-target effects [35].

IFNγ enhanced HLA-DR expression and reversed immunoparalysis in an experimental LPS model [36], in two case reports [37, 38] and in uncontrolled case series [11, 39, 40] (the largest with 18 cases). A very small trial (NCT01649921, 2 vs 2, initially planned larger) and a multicenter trial (ImmunoSep; NCT04990232) are currently unpublished.

IFNγ was effective for infection prevention in granulomatous disease [41], showed some signal for infection prevention in critically ill injured patients [42, 43] and failed to prevent hospital-acquired pneumonia (HAP) with a signal for harm in a recent trial [14].

The latter study by Roquilly administered IFNγ-1b without further stratification in critically ill patients with one or more organ failures under mechanical ventilation in a randomized placebo-controlled design to prevent HAP. It seems feasible that the recently described endotype of IFNγ/CXCL9 driven sepsis [28] could have contributed to harm from rhIFNγ-1b treatment. Consistent with harm in an endophenotypic subgroup, the authors reported in a nested exploratory study association of HAP with IFNγ-1b induced decrease in C–C motif chemokine ligand 17 (CCL17) [14]. Autoimmune diseases induced by high IFNγ levels, such as systemic lupus erythematosus have been effectively covered with IFNγ antibodies albeit with substantial variability [44] and support potential harm-mediating effects of IFNγ in the immunopathology of hyper-inflammatory sepsis. Application of IFNγ antibodies in hyper-inflammatory sepsis has not been investigated yet. Compensatory application of IFNγ recombinant protein or antibodies to modulate IFNγ activity in sepsis using HLA-DR expression to identify an immunosuppressive endotype has been studied in the ImmunoSep trial (inclusion completed, results pending) and could be included in a theranostic approach (Fig. 3).

Pharmako-kinetic behaviour of IFNγ is an important treatment variable [45]. A rapid decline of serum concentration in a monoexponentially manner after intravenous administration is reflected by a short half time of only 30 min [46, 47]. Intramuscular and subcutaneous administration as well results in protracted but fairly good absorption rate ranging between 30 to 70%, resulting in peak concentration occurring after several hours followed by measurable concentrations in a time frame of 24 h [48]. Renal elimination is held responsible for the major route of inactivation and elimination: following glomerular filtration and tubular reabsorption, interferons undergo proteolytic degradation by lysosomal enzymes, thus negligible amounts of intact and active proteins are detectable in urine [49]. Interestingly, plasma concentration of IFNγ (also of other cytokines) are only modestly influenced in septic patients undergoing continuous veno-venous haemodialysis using a 45 kDa cut-off filtration system demonstrating a small to no adsorptive capacity despite a decline in plasma cytokine concentrations [50].

In addition, hepatic deglycosylation is described as a clearance mechanism. A number of technologies have been developed to prolong the short in vivo half-life of protein drugs such as IFNγ, a few systems have been designed or intended to use for IFNγ such as controlled release formulations by minipellets or conjugation of with polyethylene glycol (i.e. PEGylation) to protect the interferon from proteolytic degradation and to reduce its renal clearance and antigenicity [45, 51–53].

In addition to the available treatment options, i.e. recombinant IFNγ application in the “immunoparalytic” endotype and treatment of the hyperinflammatory phenotype with IFNγ antibodies, signaling events downstream of IFNγ could alternatively be targeted to restore this key pathway. As illustrated in Fig. 3, JAK tyrosine kinase inhibitors, such as ruxolitinib, baricitinib and momelotinib, are potentially able to interrupt IFNγ receptor signaling and suppress the pro-inflammatory endotype of sepsis [54, 55]. Baricitinib has been successfully tested in severe COVID-19 patients [56]. Similar suppressive effects could be expected by drugs inhibiting the chemokine receptor CXCR3 including NBI 74330, AMG 487, SCH 546738 and ACT-777991 [57–59]. However, none of these inhibitors have been approved or tested for sepsis in clinical trials to date. Novel clinical studies in a theranostic design might thus pave the way for these attractive treatment options in human sepsis. Implications of IFNγ related sepsis endotypes in the clinical context and remaining questions are summarized in Boxes 1 and 2.

## Conclusions and future directions

The present literature overview reveals a central role of IFNγ in sepsis pathology and is supported by association of nucleotide polymorphisms in the IFNγ gene with endotypes of sepsis. Regarding clinical evaluation of manipulation of this signaling pathway is currently focused on recombinant IFNγ to treat sepsis-induced immunosuppression. There are promising results indicating that treatment with the recombinant protein improves host defense in the immunosuppressive phenotype of sepsis, but due to the dose-dependent ambivalent physiological and pathophysiological effects of IFNγ much work is still necessary in particular to establish which subgroups might be harmed. In contrast to the discussed efforts to restore adaptive and innate immunity in immunosuppressed sepsis patients, evidence for the benefit of IFNγ antibody therapy for the hyper-inflammatory phenotype is still missing. Due to the key importance of highly contrasting phenotypes and the dynamics of patient responses in sepsis pathogenesis, reliable diagnostics are essential prerequisites for assessing IFNγ-targeted treatment approaches in sepsis patients. In addition to established markers like HLA-DR, IFNγ-related biomarkers, such as CXCL9 or polymorphisms in the IFNγ gene, might be of potential theranostic value.

**Table:** 

BOX 1 Implications for clinical practice
– Monitoring of plasma-IFNγ in sepsis patients (in particular when part of a panel of immune effector molecules, such as HLA-DR on monocytes and chemokines) might identify endotypes amenable to immunomodulatory therapy
– Subgroups of immunosuppressed patients with sepsis might benefit from recombinant IFNγ
– Therapy with IFNγ antibodies represents a concept to treat hyperinflammatory patients expressing high IFNγ level and an “interferon-driven sepsis” endotype that has currently not been explored
– ‘Theranostic’ clinical trials that take the ambivalent role of IFNγ into consideration are warranted
– Both treatment options with recombinant IFNγ for immunosuppressed patients and IFNγ antibodies for hyperinflammatory patients need careful dose management and monitoring of clinical effects

**Table:** 

BOX 2 Open questions
– Do circulating and tissue-resident immune cells respond the same to IFNγ?
– How does IFNγ mediate hyper-inflammatory and immunosuppressive phenotypes in the pathogenesis of sepsis?
– How do IFNγ polymorphisms correlate to these phenotypes?
– How does IFNγ signaling contribute to the hyper-inflammatory phenotype and what are the treatment options?
– Are IFNγ signaling pathways, such as JAK 2 or STAT1, effective targets for therapy of hyper-inflammation and immunosuppression in sepsis?
– How does IFNγ (differentially) cooperate with other inflammatory mediators, such as the chemokines CXCL9, CXCL10 and CXCL11 and cytokines, such as IFNβ or IL-12 in the course of sepsis?

## Abbreviations

CCL: Chemokine ligand with C–C motif
CXCL: Ligand belonging to the chemokine family with a C-X-C motif
CXCR: Chemokine receptor respond to cytokines of the CXC chemokine family
HAP: Hospital acquired pneumonia
HLA-DR: Human leukocyte antigen system
HLH: Hemophagocytic lymphohistiocytosis
ICU: Intensive care unit
IDS: IFNγ-driven sepsis
IFNγ/β: Interferon-gamma/beta
IL: Interleukin
ISS: Injury severity score
JAK1, JAK2: Janus tyrosine kinases JAK1 and JAK2
LPS: Lipopolysaccharide
MALS: Macrophage activation-like syndrome
MSMD: Mendelian susceptibility to mycobacterial disease
NCT: National clinical trial
NF-κB: Nuclear factor kappaB
NK cells: Natural killer cells
NTS: Non-thyphoidal salmonella
PBMC: Peripheral blood mononuclear cells
*rh*: Recombinant human
STATs: Signal transducers and activators of transcription
TNF-α: Tumor necrosis factor alpha
UTR: Untranslated region

## Declarations

### Availability of data and materials

Data sharing is not applicable to this article as no datasets were generated or analysed during the current study.
